# Resistance to Antibacterial Agents: Foregone Conclusion - What's Next?

**DOI:** 10.1007/s12098-019-03121-0

**Published:** 2019-12-11

**Authors:** Chand Wattal, Nancy Khardori

**Affiliations:** 1 Clinical Microbiology & Immunology, Sir Ganga Ram Hospital, Rajinder Nagar, New Delhi, India; 2 Infectious Diseases, Solid Organ Transplant Program at Sentara Norfolk General Hospital, Norfolk, Virginia, USA

Resistance to antibiotics is as old as antibacterial therapy itself. The mechanisms of resistance and the implications thereof are same in all parts of the world and in all patient populations. The Indian Journal of Pediatrics invited the two editors to assemble a series of articles on the current status of resistance to antibacterial agents for its readership.

In order to put the subject matter in perspective, the first three chapters discuss information related to antibacterial agents in general. The two part review “Antibiotics: from the beginning to the future” contributed by the three US authors discusses where we are, how we got here, and what are the potential avenues to improve the gloomy prospects [1, 2]. The third article “Diagnostic microbiology from the beginning to the future: Regional antibiogram as public health tools” contributed by US authors is complementing the first two by emphasizing the role of diagnostic microbiology in management of bacterial infections [3]. The article deals with the public health relevance of microbiology and discusses in detail the generation and utilization of cumulative antibiograms at the institutional and regional levels. The pitfalls and shortcomings in large national databases on antibiotic resistance with respect to day-to-day patient care have been out into perspective. The fourth article “Controversies in treating asymptomatic bacteriuria and urinary tract infection”, also from US authors, uses a case based approach to highlight the overuse of antibiotics for a very common scenario in everyday clinical practice [4]. The next chapter by Ashok Rattan from India “How to treat sepsis in the background of resistance” focuses on the impact of pharmacodynamics and pharmacokinetics on the effectiveness of antimicrobial therapy with special reference to pediatric age groups [5].

The subsequent chapters provide data generated by authors in India. “Neonatal Sepsis part 1: Mortality and Morbidity in Neonatal Sepsis due to MDR (multidrug-resistant) organisms” is contributed by the editor from India and Neelam Kler, J K Oberoi, Anurag Fursule, Anup Kumar and Anup Thakur. Presence of multidrug resistant organisms in NNU in India is discussed in detail. The mortality due to MDRO sepsis is significantly higher as compared to infections caused by susceptible bacteria. Morbidities in neonates include prolonged use of total parenteral nutrition, need for central venous catheter, invasive ventilation, increased duration of hospital stay and neurologic sequelae [6].

“Neonatal Sepsis part 2: Treatment of Neonatal sepsis in MDRO infections” is contributed by Sankalp Dudeja and discusses all the possible therapeutic options in the background of multidrug resistance [7].

“Paediatric Intensive Care Unit Blood Culture isolates and the antibiograms generated over a period of five years” is contributed by Chand Wattal and Neeraj Goel. Specimens of blood from patients with serious infections are the most sacrosanct samples received by a bacteriology laboratory. This article adds to the scarce data from pediatric intensive care units (PICU) in India. The cumulative data reproduced here can be of use for making an antibiogram. The data clearly indicate the significant presence of MDROs in this setting [8].

The editors gratefully acknowledge the expertise and effort by the contributing authors.
